# Deep-Tissue Activation of Photonanomedicines: An Update and Clinical Perspectives

**DOI:** 10.3390/cancers14082004

**Published:** 2022-04-15

**Authors:** Nimit Shah, John Squire, Mina Guirguis, Debabrata Saha, Kenneth Hoyt, Ken Kang-Hsin Wang, Vijay Agarwal, Girgis Obaid

**Affiliations:** 1 Department of Bioengineering, University of Texas at Dallas, Richardson, TX 75080, USA; nimit.shah@utdallas.edu (N.S.); jsquire@utdallas.edu (J.S.); kenneth.hoyt@utdallas.edu (K.H.); 2 Department of Radiation Oncology, University of Texas Southwestern Medical Center, 5323 Harry Hines Blvd., Dallas, TX 75390, USA; mina.guirguis@utsouthwestern.edu (M.G.); debabrata.saha@utsouthwestern.edu (D.S.); kang-hsin.wang@utsouthwestern.edu (K.K.-H.W.); 3 Department of Neurological Surgery, Montefiore Medical Center, Bronx, NY 10467, USA; vagarwal@montefiore.org

**Keywords:** photodynamic therapy, photonanomedicines, tumor, X-ray, proton therapy, ultrasound therapy, sonodynamic therapy, two-photon, Cerenkov radiation, upconversion, bioluminescence, chemiluminescence

## Abstract

**Simple Summary:**

Photodynamic therapy (PDT) is a light-activated treatment modality, which is being clinically used and further developed for a number of premalignancies, solid tumors, and disseminated cancers. Nanomedicines that facilitate PDT (photonanomedicines, PNMs) have transformed its safety, efficacy, and capacity for multifunctionality. This review focuses on the state of the art in deep-tissue activation technologies for PNMs and explores how their preclinical use can evolve towards clinical translation by harnessing current clinically available instrumentation.

**Abstract:**

With the continued development of nanomaterials over the past two decades, specialized photonanomedicines (light-activable nanomedicines, PNMs) have evolved to become excitable by alternative energy sources that typically penetrate tissue deeper than visible light. These sources include electromagnetic radiation lying outside the visible near-infrared spectrum, high energy particles, and acoustic waves, amongst others. Various direct activation mechanisms have leveraged unique facets of specialized nanomaterials, such as upconversion, scintillation, and radiosensitization, as well as several others, in order to activate PNMs. Other indirect activation mechanisms have leveraged the effect of the interaction of deeply penetrating energy sources with tissue in order to activate proximal PNMs. These indirect mechanisms include sonoluminescence and Cerenkov radiation. Such direct and indirect deep-tissue activation has been explored extensively in the preclinical setting to facilitate deep-tissue anticancer photodynamic therapy (PDT); however, clinical translation of these approaches is yet to be explored. This review provides a summary of the state of the art in deep-tissue excitation of PNMs and explores the translatability of such excitation mechanisms towards their clinical adoption. A special emphasis is placed on how current clinical instrumentation can be repurposed to achieve deep-tissue PDT with the mechanisms discussed in this review, thereby further expediting the translation of these highly promising strategies.

## 1. Photonanomedicines and Current Clinical Practice

### 1.1. Photodynamic Therapy and Photonanomedicines

Photodynamic therapy (PDT) is a light-based treatment modality that exhibits unparalleled spatiotemporal control over tumor tissue phototoxicity and photodynamic priming for synchronized combination regimens. It does so by combining nonthermal light, non-toxic and wavelength-specific photosensitizer (PS) molecules, and often molecular oxygen to generate a number of cytotoxic and biomodulatory reactive molecular species (RMS) [1]. RMS primarily include singlet oxygen (^1^O_2_) in addition to hydroxyl radicals (^•^OH), superoxide anion (O_2_^−^^•^), peroxyl radicals (ROO^•^), nitrogen-based radicals, and biological radical intermediates [1]. Its capacity to induce tumor tissue destruction and modulation in a manner that is non-overlapping with secondary modalities distinguishes PDT-based combination regimens and provides a safer option for synergy. Furthermore, PDT’s non-overlapping mechanisms of action with subcellular, cellular, vascular, and stromal control over modulation has resulted in PDT being adopted as a widely enabling technology for a plethora of clinically used and emergent therapeutic regimens. To that end, nanotechnology has played a central role in facilitating PDT-based combination regimens, whereby the formulated nanomedicine platform serves as both a delivery system and a phototriggered release system when activated for PDT. Photoactivable nanomedicines that incorporate PSs or photocatalytic (nano)materials are referred to as photonanomedicines (PNMs) [2,3]. The first PNM approved in the year 2000 for wet age-related macular degeneration was Visudyne, a lipid nanoformulation of the PS benzoporphyrin derivate (BPD). Since then, various interactions of similar PNMs have evolved to include a plethora of primarily hydrophobic PS molecules and photocatalytic nanomaterials [1,4,5]. In addition, incorporation of secondary and tertiary therapeutics, such as small molecular weight inhibitors, immunotherapeutics, and chemotherapeutics, have pushed the boundaries of conventional PNMs towards spatiotemporally synchronized multifunctionality [1,5,6].

A uniting facet amongst the majority of these PNMs is their amenability to photoactivation with light spanning the ultraviolet, visible, and near-infrared regions of the electromagnetic spectrum. PS molecules excited by shorter wavelengths of light are beneficial in confining phototoxicity to superficial lesions while protecting deeper cell layers. A recent clinical example includes TLD-1433, a ruthenium-based PS that is in Phase 2 trials for non-muscle invasive bladder cancer (NCT03945162), which broadly absorbs blue, green, and red light [7]. The depth of tissue photodamage is thus controlled by the wavelength of light used for excitation. More commonly, for deeply seated solid tumors, PS molecules are excited by light within the tissue optical window (far red to near-infrared wavelengths). These include 750 nm (redaporfin), 690 nm (BPD and IRDye700DX), and 670 nm (chlorin e6).

In the clinic, light dosimetry for PDT of a deeply seated tumor is typically more complex than PDT of surface tumors. As such, sophisticated strategies for photoexcitation are needed to ensure that homogenous PS and PNM activation is achieved and that complex light interactions within solid tumors are carefully accounted for. In addition, successful photoactivation requires the accurate identification of tumor margins and distant metastases, given that they are all accessible to the light sources used in the clinic. These requirements and challenges have resulted in the emergence of novel approaches to activate PNMs deep within the body with alternative energy sources that use direct and indirect mechanisms for activation. These will be discussed in greater detail in Section 2.

**Figure 1:**
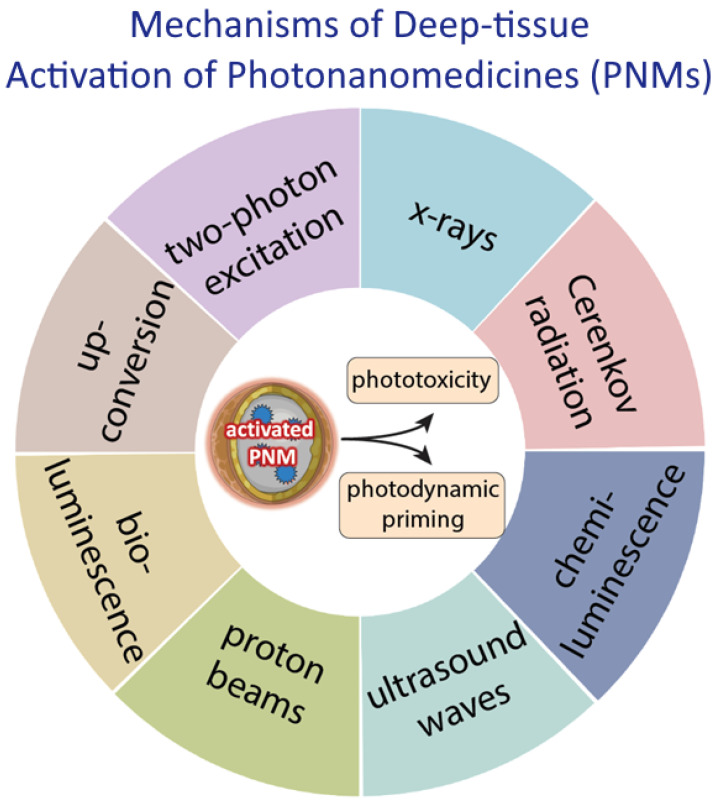
A summary of various mechanisms of deep-tissue activation of photonanomedicines (PNMs) using alternative tissue permeating energy sources that will be discussed in this review. Deep-tissue activation of the PNM leads to phototoxicity or photodynamic priming of the tumor microenvironment that can enable and potentiate combinatorial modalities. (Figure is created from Biorender.com using an Academic License).

Direct deep-tissue PNM activation mechanisms typically involve alternative, deeply penetrating energy sources that interact directly with specialized nanomaterials used within the PNM. These direct interactions result in the emission of photons to activate associated PS molecules through Forster resonance energy transfer (FRET), or in electronic excitation within the nanomaterial crystal lattice that initiates a cascade of cytotoxic and biomodulatory chemical reactions at the electron-hole loci. Examples of direct deep-tissue activation mechanisms include: (1) upconversion (the conversion of infrared light into shorter wavelength visible light capable of PNM activation); (2) two-photon excitation (the excitation of visible light activable PNMs using two long wavelength photons simultaneous); (3) scintillation (the conversion of ionizing radiation into shorter wavelength visible light capable of PNM activation); and (4) radiosensitization (the electronic excitation of PNM photocatalysts or PSs embedded in PNMs using ionizing radiation). Indirect deep-tissue PNM activation mechanisms typically involve biochemical and/or mechanochemical processes, such as bioluminescence, chemiluminescence, and ultrasound-induced cavitation, or secondary photon generating phenomena, such as Cerenkov radiation, in response to radionuclide decay or high energy external beam radiation passing through tissue. The direct and indirect deep-tissue PNM activation mechanisms that have been used to induce tumor tissue phototoxicity or photodynamic priming for secondary or tertiary modalities are summarized in Figure 1.

The photonically active materials used for PNMs capable of deep-tissue PDT vary significantly in composition and nanostructure. Most of these emergent materials are experimental and have not been used extensively in humans to date. However, a number of the constituents of these photonically active materials are currently in clinical trials for other applications, thereby demonstrating some clinical potential as integral components of deep-tissue-excitable PNMs (Table 1).

### 1.2. Current Clinical Irradiation Strategies for PDT 

Currently, clinical PDT of non-superficial tumors is mostly performed with far red or near-infrared light activable sensitizers, whereby light is delivered by light diffusing balloons or interstitial fibers, with and without image guidance. The greatest clinical impediment to PDT remains its complex dosimetry. While this is also a challenge for radiotherapy, innovative strategies, such as dose painting with intensity-modulated radiation therapy (IMRT) techniques, have been developed to address some of these challenges in heterogeneity in tumor oxygenation. With respect to PDT, dosimetry is more complex than for radiation therapy, as homogenous tumor tissue distribution of the PS or PNM is also critical, in addition to sufficient oxygenation for oxygen-dependent PS agents and sufficient light distribution to achieve threshold levels of therapeutic RMS.

#### 1.2.1. Interstitial PDT 

A number of PDT irradiation protocols have been developed for non-superficial lesions and disseminated lesions, for which simple externally applied irradiation is not appropriate. These are primarily interstitial diffusing tip fibers inserted into the bulk of the tumors, also known as interstitial PDT (I-PDT), in addition to light diffusing balloons that fill irregular lesion-containing cavities to enable homogenous irradiation. Interstitial PDT (I-PDT) is used for deep-seated or locally advanced tumors that are greater than or equal to 10 mm in thickness [8] During I-PDT, the tissue localized PS molecules or PNMs are typically activated with laser light or LED light delivered through diffusing tip optical fibers which are further inserted into tumors through sterilized catheters [9]. I-PDT has been approved in Europe for esophageal cancer using temoporfin and in the US for esophageal cancer and other malignancies using Photofrin [9]. In the clinic, activation of the PS Photofrin is performed using 630 nm laser light and power density of 400 mW/cm which is delivered through diffusing tip optical fibers. Typical energy densities used are between 50 to 300 J/cm with respect to the length of diffuser [10]. In another example of I-PDT in patients with head and neck cancer, a single dose of 0.15 mg/kg Temoporfin is administered by slow intravenous injection into the antecubital vein in no less than 6 m. Laser light of 652 nm wavelength is delivered through a diffusing tip fiber at a dose of 20 J/cm, and an irradiance of 100 mW/cm (NCT01415986). In another study of lung carcinoma, 2 mg/kg of porfimer sodium was injected 48 to 50 h prior to light illumination. A cylindrical laser fiber was used through a guide sheath to the tumor to deliver light for photoirradiation (NCT04753918). 

I-PDT has also been performed on patients with locally advanced pancreatic cancer. In a study by Huggett et al., patients (*n* = 15) were treated with a dosage of 0.4 mg/kg of the PNM Visudyne through intravenous injection [11]. The tumor was then punctured using a 19-gauge needle positioned percutaneously under computed tomography (CT) guidance. Next, an optical fiber with a 0.4 mm diameter along with 1 cm diffuser tip was inserted through the needle. After 60 to 90 min of Visudyne administration, laser light with a 690 nm wavelength and 0.3 W of power (150 mW/cm irradiance) was used for irradiation. Out of 15 patients, 13 patients were treated with a single fiber and a light dose of 5 J at 150 mW/cm for 33 s. The light dose was doubled with a maximum of 40 J until the desired level of necrosis was achieved. Two other patients were treated with two fibers and three fibers, respectively. Results indicated that there was no sign of necrosis when treated with 5 J. Furthermore, at 10, 20, and 40 J, necrosis was seen with a mean volume of 0.46, 1.14, and 3.48 cm^3^, respectively. This suggests that I-PDT using the PNM Visudyne is a viable and safe option for treating patients with pancreatic adenocarcinoma as it has low adverse effects with a significant induction of tumor necrosis.

In another study, Henada et al. performed endoscopic ultrasound (EUS)-guided PDT using the PNM Visudyne for locally advanced pancreatic cancer [12]. The main objective was to assess the safety and efficacy of treatment with EUS guidance. The authors used 0.4 mg/kg Visudyne administered through intravenous injection in patients (*n* = 8) with pancreatic cancer. For EUS the authors used an ultrasound gastrovideoscope (UCT 180) with a console (F75) for guiding a 19-gauge needle inside the tumor. An optical fiber with a 0.4 mm diameter was used with a 1 cm long diffuser tip for delivering 690 nm light with an irradiance of 150 mW/cm for 333 s. The light was delivered after 60 to 90 min of Visudyne injection. Results demonstrated necrosis of tumors with a mean diameter of 15.7 ± 5.5 mm in five patients with an initial tumor volume of 33.3 ± 13.4 mm after 48 h. EUS was effective at helping visualize the real-time positioning of the needle inside the tumor and reduced off-targeting. 

A similar clinical study was carried out by DeWitt et al. to assess the safety and efficacy of EUS-guided PDT for locally advanced pancreatic cancer using the PS porfimer sodium at a dose of 1 or 2 mg/kg [13]. After 2 d, EUS-PDT was performed on patients using a 19-gauge needle by inserting a light diffuser of 1 cm in length. The authors used 630 nm wavelength light with a power of 400 mW. The time duration was 125 or 250 s. Results showed a reduction in tumor volume by 10 ± 26 cm^3^ and 18 ± 22% necrosis. The authors concluded that this EUS-guided I-PDT approach can be a safe and efficient treatment modality for locally advanced pancreatic tumors.

#### 1.2.2. PDT of Disseminated Disease

PDT of disseminated malignancies in the clinic often requires sophisticated light delivery applications to provide homogenous irradiation of the lesions. In a study on patients with malignant pleural mesothelioma, two different surgical techniques, along with intraoperative PDT, were performed on 28 patients and compared [14]. The authors carried out macroscopic resection using two methods: one was modified extrapleural pneumonectomy (MEPP) (*n* = 14) and the other was radical pleurectomy (RP) (*n* = 14). Both groups were treated with intraoperative PDT using 0.01% intralipid as a light diffuser filling the chest cavity. Patients were given a porfimer sodium dosage of 2 mg/kg at 24 h before surgery. The authors used 630 nm laser light with a dose of 60 J/cm^2^ for approximately 1 h. Light dosimetry was determined using a custom dosimetry system. The results indicated that apart from the advantage of saving the lung, RP+PDT gave a higher survival period of 2.1 years (y) compared to MEPP+PDT with a survival period of 8.4 months (mo). This suggests that RP+PDT using intralipid light dispersant for homogenous irradiation is a superior surgical option in patients with malignant pleural mesothelioma. 

In another completed clinical trial on pediatric patients with brain tumors (NCT01682746), PDT was performed on the surgical bed after tumor debulking. Patients were injected with photofrin (2 mg/kg) before surgery and PDT was performed using a wavelength of 630 nm and a total power of 240 J/cm^2^. An optical fiber was placed inside the surgical cavity after the removal of tumor. Intralipid was then inserted into the cavity through a light diffusing balloon. This helped to diffuse the light within the cavity for homogenous irradiation of residual unresectable tumor cells. 

While these strategies have demonstrated a clinical benefit for the photoirradiation of focal and disseminated tumors, there is an impetus to harness alternative energy sources for the activation of PS and their respective innovative PNMs deep within the human body. In doing so, PNM activation procedures move towards becoming entirely non-invasive and help enable deep-tissue PDT of irregular lesions and metastatic sites. These technologies will be described in detail in Section 2. 

## 2. State of the Art of Deep-Tissue Activation for PDT

As discussed above, deep-seated tumors require sophisticated clinical irradiation approaches in order to fully irradiate tumors, especially those with complex geometries. Moving forward, the field is adopting various strategies for deep-tissue activation of PSs and their respective PNM formulations whereby noninvasive and innovative activation approaches for PNMs are harnessed. PNM activation can thus be simplified for deeper tumors and can be achieved readily and more completely in disseminated and metastatic lesions. Noteworthy advances in state-of-the-art activation for deep-tissue PDT include two-photon PDT, upconversion PDT, X-ray induced PDT, Cerenkov-radiation-induced PDT, luminescence (CRET and BRET)-mediated PDT, and ultrasound-mediated PDT (sonodynamic therapy and derivatives).

### 2.1. Two-Photon PDT

**Figure 2:**
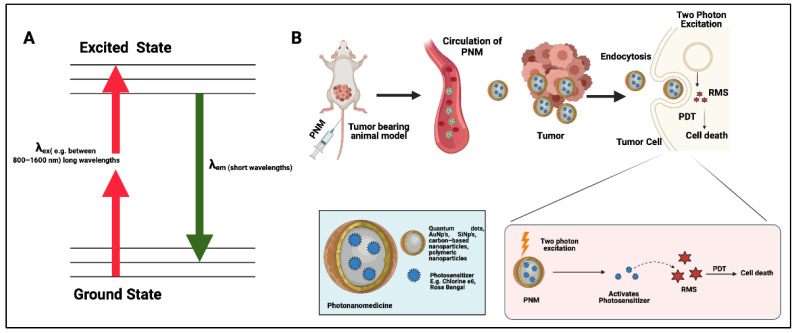
Schematic representation of two-photon-mediated photodynamic therapy (PDT). (**A**) A Jablonski diagram of two-photon excitation which shows how PNM can be activated using two-photon excitation (TPE). (**B**) The administration of PNMs into the animal model which accumulate at the tumor site. When activated by TPE, they generate reactive molecular species (RMS) which cause biomodulation and ultimately cell death. (Figure is created from Biorender.com using an Academic License.)

The concept of two-photon excitation is frequently used for imaging deeper in tissue. The process involves two photons of longer wavelengths of light (usually between 800 nm and 1600 nm) which are used to simultaneously excite a chromophore that typically absorbs shorter wavelengths of light approximately half the wavelength used for excitation. In doing so, longer wavelengths of light that preferentially penetrate tissue within the optical window become capable of exciting exogenous or endogenous chromophores at 10- to 100-fold greater tissue depths [15]. Although widely adopted for tissue imaging, some evidence exists for two-photon-excited deep-tissue PDT. In these applications, either a PS formulated into PNMs, or optically active nanoparticles (FRET donors for associated PS molecules) are directly excited using the two-photon process. PNMs that have been demonstrated to serve as efficient platforms for two-photon PDT include quantum dots [16], silica nanoparticles [17], gold nanoparticles [18], graphene carbon-based nanoparticles [19], and polymeric nanoparticles [20]. Although some preclinical in vitro and in vivo data has shown promise, a significant limitation of two-photon PDT is the high photon density required for the simultaneous excitation using two photons. This can only be achieved using focused laser light which serves to activate PNMs only within a focal volume of several μm^3^ [21]. Given this extremely limited focal volume of excitation, the practicality of performing two-photon PDT for large and disseminated lesions in vivo remains in question. Figure 2**.** shows a schematic representation of the mechanisms underlying two-photon PDT as well as the process of generation of therapeutic reactive molecular species.

Zhao et al. demonstrated that gold nanorods (AuNRs) with three different aspect ratios could serve as two-photon PDT agents without the need for additional organic PS molecules [15]. Although AuNRs exhibit low ^1^O_2_ quantum yields (0.1%), the two-photon absorption cross-sections are of the order of 10^8^-fold higher than organic PSs, such as rose bengal (RB). As such, they have been shown to generate ^1^O_2_ in quantities sufficient for cell killing upon two-photon excitation. The AuNRs in this study were coated with polyvinyl pyrrolidone (PVP) for improved uptake within cells and to increase biocompatibility. The efficacy of using three different wavelengths of two-photon excitation (765, 808, and 865 nm of pulsed laser light) was compared in HeLa cell lines. AuNRs reduced cell viability to 73.2, 32.8, 26.7, and 18% when irradiated with an 808 nm femtosecond laser light for 3, 6, 10, and 15 min, respectively. There was negligible photokilling of cancer cells when treated with RB, even after 15 min irradiation. However, considering that AuNRs are also highly efficient photothermal agents and the high irradiances typically associated with Ti:sapphire femtosecond lasers (W/cm^2^ range), it is difficult to rule out the contribution, or even the predominance, of photothermal therapy (PTT) in this study. 

Gao et al. developed a dual approach of combining two-photon PDT with PTT using PS-coated gold nanocages [22]. The authors developed a nanocluster for two-photon therapy using hypocrellin B coated with lipid and spread this layer onto the gold nanocages (AuNC). The authors used 790 nm irradiation laser light for two-photon therapy. In vitro analysis was carried out on HeLa cells using the MTT assay which showed higher cell viabilities of approximately 90% for control groups (cells, no agents, with irradiation; cells, with agents, no irradiation; and untreated cells). Significantly lower viability (17.4 ± 4.3%) was observed in cells treated with the nanocluster complex (35 pM AuNCs and 7.0 μM hypocrellin B) combined with two-photon excitation at 790 nm (85.5 pJ per pulse for around 5 min). Thus, this combination of a PS and an AuNC photothermal transducer, along with two-photon laser excitation, is capable of synergistic cancer therapy.

Secret et al. developed porous silica nanoparticles and conjugated them with porphyrin molecules to demonstrate two-photon excitation for PDT [17]. Cytotoxicity experiments were carried out using an MTS assay on MCF-7 breast cancer cell lines. Higher cytotoxicity was seen in the cells when treated with silica-porphyrin complex and two-photon irradiation with 800 nm light (3 scans for 1.57 s) with a cell viability of 25%, while the control groups remained at a cell viability of >80%.

### 2.2. Upconversion PDT

**Figure 3:**
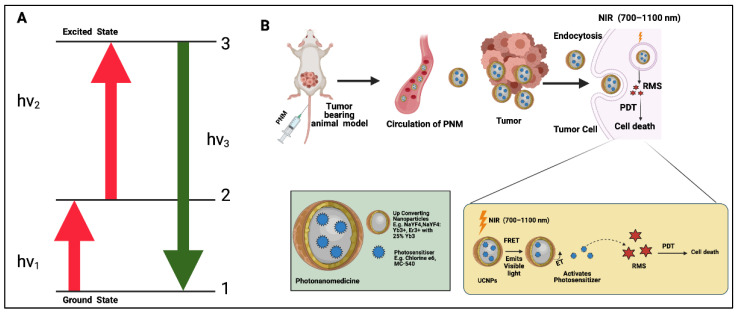
Schematic representation of general mechanism of upconversion PDT. (**A**) A Jablonski diagram demonstrating the upconversion process whereby two consecutive long wavelength excitations (hv_1_ and hv_2_) of upconverting nanoparticles (UCNPs) result in short wavelength emission (hv_3_) from higher energy levels. (**B**) Administration and circulation of UCNP-based PNMs within the blood vessels and their uptake in cancer cells. Furthermore, the general mechanism of cell killing through production of reactive molecular species is demonstrated. UCNPs are activated by NIR light which further emits visible light and excites PSs for upconversion PDT. (Figure is created from Biorender.com using an Academic License.)

Treating tumors with higher depths becomes difficult using external light sources during conventional PDT because of the limited penetrance of visible light [23]. Near-infrared (NIR) light ranges from 700 nm to 1100 nm [24]. This is also the window of optical transparency for tissues, which is frequently used for two-photon excitation as described above. Another avenue that leverages NIR light excitation is upconversion PDT, where upconverting nanomaterials are used as a light source for deep-tissue PDT [25]. These upconverting nanoparticles (UCNPs) can convert NIR light into visible photons and help to trigger PSs, most commonly through resonance energy transfer [26]. An example of UCNPs used for upconversion PDT includes lanthanide-doped nanoparticles (NaYF_4_:Yb^3+^, Er^3+^ with 25% Yb^3+^, NaYF_4_:Yb^3+^/Er^3+^/Gd^3+^, and NaYF_4_). Figure 3 is a schematic representation of the processes underlying upconversion PDT, along with the subsequent process of generating reactive molecular species.

**Figure 4:**
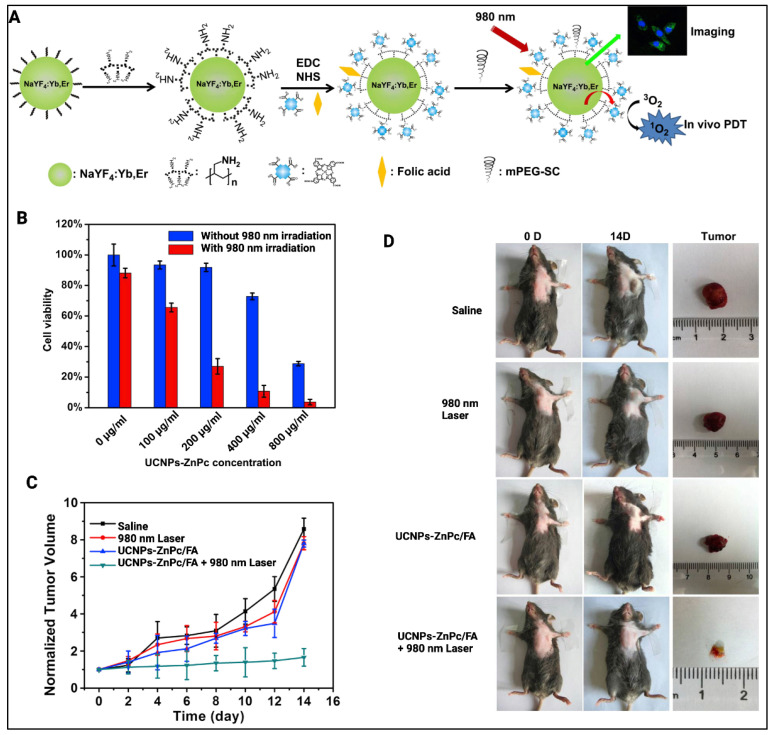
(**A**) Synthesis stages of UCNPs-ZnPc constructs prepared by Xia et al. (**B**) Cell viability of HeLa cells treated with and without irradiation (0.39 W/cm^2^ at 980 nm) along with different concentration of UCNPs-ZnPc/FA. (**C**) Hepa1-6 tumor volumes in different groups of treatment including upconversion PDT. (**D**) Images of tumor and mice in the same treatment groups as in (**C**). Reprinted and adapted with permission from [27]. Copyright (2014) American Chemical Society.

As shown in Figure 4, Xia et al. developed UCNPs using zinc phthalocyanine (ZnPc) as a PS [27]. The authors doped lanthanides (Yb^3+^, Er^3+^) into a NaYF matrix along with ZnPc for transfer of energy and coated the construct with folic acid (UCNPs-ZnPc/FA) for cancer cell targeting. In vitro analysis was carried out on HeLa cells through MTT assay. The cells were incubated with UCNPs-ZnPc/FA and then irradiated with 980 nm laser light (0.39 W/cm^2^). Different concentrations (0, 100, 200, 400, and 800 μg/mL) of UCNPs-ZnPc/FA were used to assess the concentration dependent upconversion PDT effect. The authors also performed in vivo experiments using female mice (*n* = 6) with Hepa1-6 tumors (20 g, 6 to 8 w). Mice were then divided into four groups: one group with saline as the control, one group with only irradiation, one group with only UCNPs-ZnPc/FA, and one group with UCNPs-ZnPc/FA combined with irradiation through a 980 nm laser light (0.39 W/cm^2^) for 15 min. Results indicated significant inhibition of tumor growth in the group treated with UCNPs-ZnPc/FA and irradiation with the 980 nm laser light. The tumor inhibition ratio was about 80.1% when compared to the control group with only saline. With a similar approach, Idris et al. developed NaYF_4_ UCNPS and coated them using mesoporous silica [28]. Along with this, the authors used two different PSs, merocyanine 540 and zinc phthalocyanine, and irradiated these UNCPs with a 980 nm laser. Excitation resulted in the emission of multiple visible light peaks which helped activate both PSs. In vitro experiment showed around 45% cell viability when treated with ZnPc/MC540-UCNP along with 980 nm light irradiation (2.5 W/cm^2^) for 40 min. In vivo studies revealed reduced tumor volumes of around 150 mm^3^ when treated with ZnPc/MC540-UCNP along with 980 nm light irradiation (2.5 W/cm^2^) compared to the control group with a volume of 1200 mm^3^. 

Cui et al. developed a multifunctional nanoconstruct using UCNPS with a zinc phthalocyanine PS [29]. The authors coated the constructs with folate-modified amphiphilic chitosan to facilitate tumor targeting of the folate receptor that is frequently overexpressed in a number of cancers. In vivo experiments were carried out using mice with S180 tumors (*n* = 8). They were treated with UCNP-ZnPc with folate coating and activated with 980 nm laser light (0.2 W/cm^2^ for 30 min). Upconversion PDT demonstrated an approximately 50% tumor inhibition ratio. Wang et al. also developed NaFY_4_-based UCNP along with the PS chlorin e6 and coated them with polyethylene glycol [26]. In vitro results on HeLa cells showed a cell viability of around 20% in the group treated with UCNP-Ce6 in combination with the 980 nm laser (0.5 W/cm^2^) while groups treated separately with UCNP alone or Ce6 alone exhibited more than 85% viability after treatment. For the in vivo studies, female Balb/c mice with 60 to 70 mm^3^ tumor volumes were used. Results showed a six-fold lower tumor volume when treated with UCNP-Ce6 (20 mg/mL UCNPs and 1.5 mg/mL Ce6) with a 980 nm laser (0.5 W/cm^2^) for 30 min compared to the saline group.

While highly promising, the potential toxicity concerns of UCNPs will undoubtedly delay clinical translation, even though they are still worth pursuing. While the components of UCNPs may not necessarily be inherently toxic, the nanocrystalline composition of the components, their morphology, aspect ratio and size may all impact their physiological clearance rates, toxicity, ability to cross the blood-brain barrier and interaction with nuclei [30].

### 2.3. X-ray PDT 

**Figure 5:**
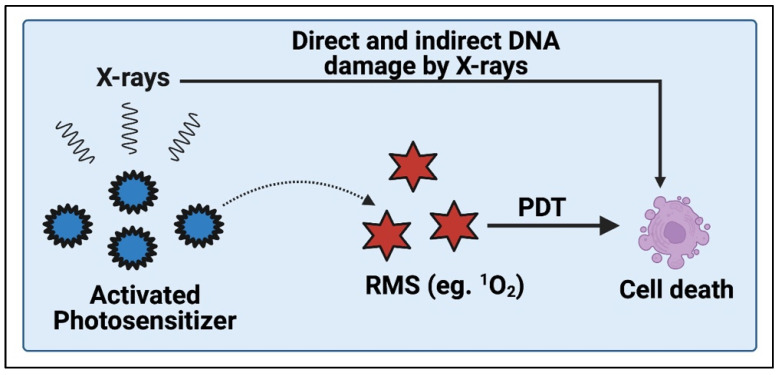
Schematic representation of the general mechanism of action for direct X-PDT without the use of a nanoscintillator intermediate. (Figure is created from Biorender.com using an Academic License.)

**Figure 6:**
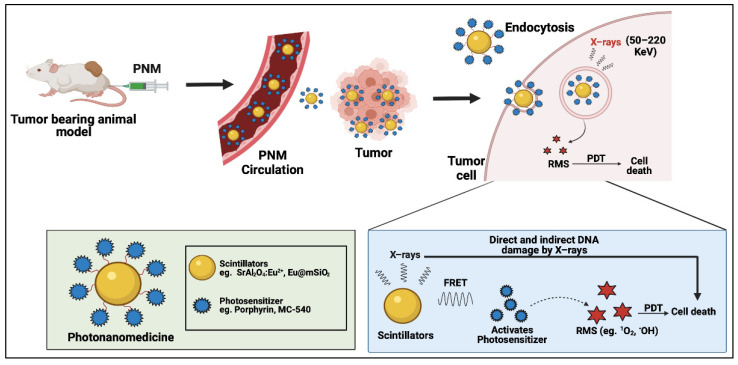
A schematic diagram that shows the general mechanism of nanoscintillator facilitated X-PDT. The figure represents the administration of nanoscintillator-based PNM into the animal model and its uptake in tumor tissue. Furthermore, it demonstrates the general X-PDT process using nanoscintillator systems as fluorescence resonance energy transfer (FRET) donors for the generation of reactive molecular species (RMS). (Figure is created from Biorender.com using an Academic License.)

X-rays are ionizing electromagnetic waves of photons that have been historically used in radiation therapy for cancer. Radiation therapy using X-rays has the advantage of readily penetrating soft tissue with the use of external beam linear accelerators (LINACs) [31]. The energy used in clinical radiation therapy is in the range of keV or MeV. In recent years, X-rays of varying energies have been explored for their ability to activate PSs deep within tissue, thereby inducing X-ray PDT (X-PDT) in the absence of visible or NIR light activation. Unlike conventional photoexcitation of PSs, X-ray excitation is an inefficient process that likely involves Coulombic excitation and ejection of electrons [32]. Studies have shown that intracellular protoporphyrin IX can be directly excited with X-rays to generate ^1^O_2_ and induce tumor cell death [33,34,35]. Figure 5 is a schematic representation of direct X-PDT. Considering the low efficiency of X-PDT, the process often requires the use of a nanoparticle intermediate that, upon excitation with X-rays, emits visible light that activates an attached PS. These types of nanoparticle transducers are called scintillators and the phenomenon is called X-ray-excited optical luminescence (XEOL) [32]. After getting excited by X-rays, these scintillating nanoparticles transfer energy to the PS which in turn excites them and results in the production of therapeutic and biomodulatory RMS. This specific form of X-PDT is also referred to as radiodynamic therapy [32,36]. Apart from RMS, the direct or indirect cellular action of ionizing X-ray radiation can also contribute to cell death as part of a conventional radiotherapy regimen. In these instances, X-rays can directly damage DNA or can result in damage to DNA, lipids and proteins by the production of water-based and oxygen-based radicals [37]. Different types of scintillating nanoparticles have been explored in this context, such as GdEuC1_2_, SrAl_2_O_4_:Eu^2+^, and Tb_2_O_3_, amongst many others. Figure 6 shows a schematic representation of the mechanism of X-PDT using scintillating nanoparticle systems. Table 2 shows the range of different energies of X-rays used, along with the different sensitizers that have been used for X-PDT. Rossi et al. reported on the X-PDT activity of porphyrin-conjugated nanowires using low doses (0.4–2 Gy) of high energy 6 MeV X-rays delivered using a clinical Varian Clinac LINAC system [38]. A tetracarboxyphenyl porphyrin derivative PS was conjugated to inorganic SiC/SiOx core/shell nanowires through click chemistry and incubated with A549 adenocarcinomic human alveolar basal epithelial cells for 24 h. At 12 d following irradiation with 0.4–2 Gy of 6 MeV X-rays, clonogenicity was assessed. X-PDT using the porphyrin-conjugated nanowires reduced cell clonogenicity by 75% with respect to irradiated cells in the absence of A549 cells, thereby demonstrating that high-energy, clinically relevant 6 MeV X-rays can serve as effective activators for X-PDT. Although without the use of a PNM, another study using clinically relevant 4 MeV X-rays delivered using a clinical PRIMUS Mid-Energy LINAC system demonstrated that X-PDT was again feasible at X-ray energies higher than the typical keV ranges used for pre-clinical activation of PNMs [39].

**Figure 7:**
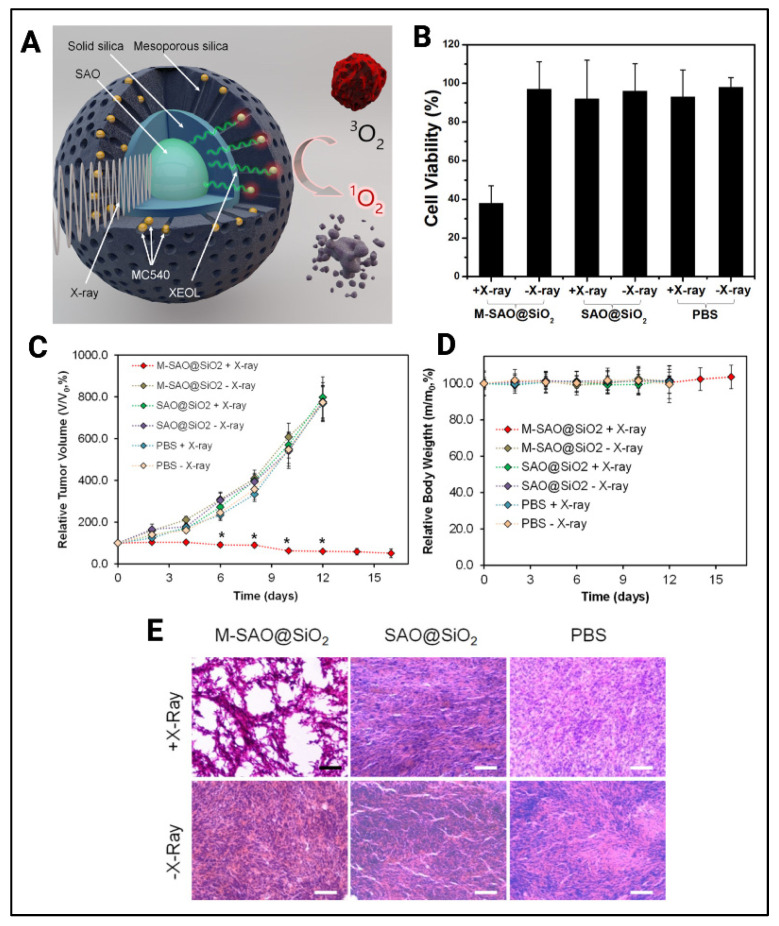
(**A**) Schematic of X-PDT using a SrAl_2_O_4_:Eu^2+^ nanoscintillator with the PS MC540. (**B**) Cell viabilities of U87MG (human glioblastoma) with treatments at different groups with and without X-rays and nanoscintillators (*n* = 4). (**C**) U87MG tumor growth curves following X-PDT (V/V0%, *n* = 5). (**D**) Changes in animal body weight following X-PDT. (**E**) H&E staining of tumor tissues following X-PDT. Reprinted and adapted with permission from [36]. Copyright (2015) American Chemical Society.

As mentioned above, nanoscintillators have served as critical transducers that potentiate X-PDT. Chen et al. used a SrAl_2_O_4_:Eu^2+^ nanoscintillator coated with solid silica followed by a second outer layer of mesoporous hollow silica (Figure 7) [36]. The PS merocyanine 540 was embedded between the two silica layers. This nanoscintillator was activated using 50 keV X-ray irradiation (0 to 5 Gy) which then excited the PS following scintillation. This PS can further produce cytotoxic ^1^O_2_, measured using the fluorescent singlet oxygen sensor green (SOSG) probe. Results showed an increase in fluorescence of the SOSG probe when exciting the MC540-SAO:Eu@mSiO_2_ using X-ray irradiation, compared to MC540-SAO:Eu@mSiO_2_ without excitation. The authors performed an MTT assay to assess the X-PDT response in U87MG cells. After treating the cells with MC540-SAO:Eu@mSiO_2_ (50 μg/mL), and irradiating using 1 Gy/h for 30 min, cell viability was measured and found to be 62.0 ± 9.0%. In vivo studies were carried out in U87MG tumor-bearing mice (*n* = 5). The authors found that in animals treated with MC540-SAO:Eu@mSiO_2_ (4.25 mg/kg) and 50 keV X-rays (1 Gy/h for 30 min), tumor growth was arrested at 12 d and average tumor volume was reduced to 60.2 ± 6.9%. In contrast, the control groups exhibited a rapid and distinguishable increase in tumor growth. 

In another noteworthy study, Bulin et al. used terbium oxide nanoscintillators with silica oxide shells for X-PDT [49]. The authors grafted this core nanoparticle with the PS porphyrin and quantified the X-ray induced production of ^1^O_2_ using two commercially available probes: SOSG and 3′-p-(aminophenyl) fluorescein (AFP). The results suggested that the group with the nanoscintillator complex Tb_2_O_3_@SiO_2_ conjugated to the porphyrin generated greater amounts of ^1^O_2_ compared to the porphyrin alone following irradiation with X-rays.

Clement et al. developed a polymeric nanoparticle construct with the PS verteporfin and the transactivator of transcriptome (TAT) peptide for nuclear localization [50]. The size of the construct was 85 nm, and the zeta potential was +2.3 mV. To assess the efficacy of the X-PDT, the authors performed live/dead cell viability assays on human pancreatic cancer cells (PANC-1). The results showed that cells with an individual treatment of X-rays (4 Gy) exhibited 90% cell viability. However, cells treated with X-rays and the PLGA-VP-TAT PNM exhibited <50% cell viability. Cell viability results were found to be consistent with the ^1^O_2_ generation when measured using the SOSG probe. Fluorescence signals were found to be less than 5% in the cells treated with X-ray alone, while the cells treated with PLGA-VP-TAT exhibited a 280% increase in SOSG signal, compared to untreated cells. Double-stranded DNA breaks measured using γ-H2AX revealed a six-fold increase in double-strand DNA breaks when treated with X-PDT compared to the control group. The authors also reported a survival fraction of <0.1 for cells treated with X-PDT as measured by clonogenicity assays.

It is worth noting that other electron-dense metallic nanoparticles and PS-free nanoscintillators have also been used to potentiate radiation therapy by radiosensitization, although these mechanisms are distinct from X-PDT [51,52]. The mechanism of radiosensitization with such nanoscintillators and metallic nanoparticles center on localized radiation therapy dose deposition [53,54]. Gold nanoparticles are the most widely used radiosensitizing nanoparticle that, upon X-ray excitation, deposit X-rays locally and eject high energy Auger and Compton electrons that can be used for therapy. Recent seminal work by Bulin et al. also found that nanoscintillators that are void of PSs are still potent therapeutic agents when activated by X-rays by acting as radiation dose enhancers [38,55]. As such, it is conceivable that radiation dose enhancement is a prominent phenomenon that contributes to the efficacy of X-PDT that leverages PSs and therapeutic RMS.

While highly promising, the true clinical potential of X-PDT is likely to be realized using clinically relevant X-ray energies. It is evident from the literature that the vast majority of X-ray energies that have been used preclinically for X-PDT are within the keV range. Studies using MeV X-ray energies for X-PDT have been limited. The primary reason is that X-ray irradiators available for preclinical research are largely limited to sub-320 keV energies. These, however, are not clinically relevant as linear accelerators (LINAC) used in the clinic typically operate in the MeV energy range. As such, X-PDT would need to either configure clinic X-ray irradiators or leverage X-PDT technologies that can also be activable by X-rays in the MeV range, such as the study by Rossi et al. [39]. The applicability of current clinical X-ray sources will be discussed in more detail in Section 3.

### 2.4. Cerenkov-Radiation-Induced PDT

**Figure 8:**
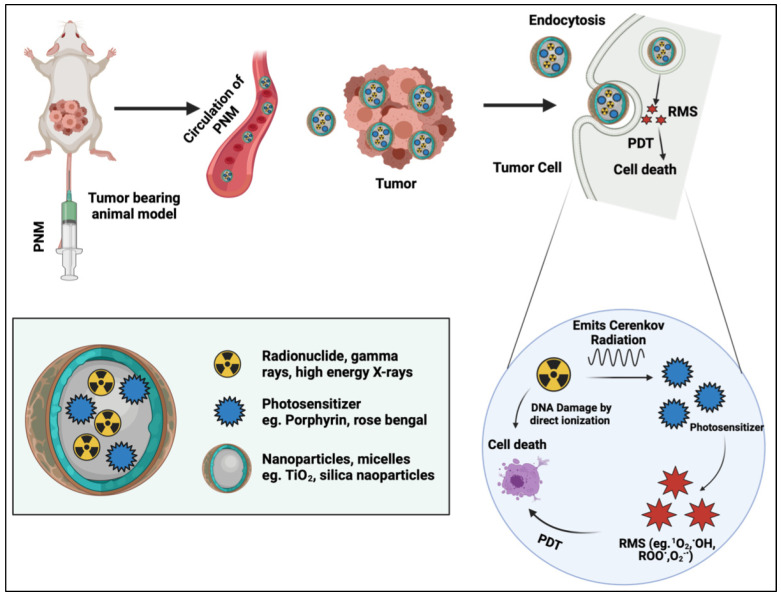
Schematic representation of Cerenkov-radiation-induced PDT represents the injection of a PNM system into the animal model and its accumulation in tumors. Furthermore, it represents the process of generating reactive molecular species through activation of a PS by Cerenkov radiation, ultimately leading to cell death. (Figure is created from Biorender.com using an Academic License).

Cerenkov radiation is the emission of broad-spectrum visible light when a charged particle or photon travels faster than the phase velocity of light through a dielectric medium. Cerenkov radiation has been used as an internal photon source to activate PSs deep within the body without the need for external photoactivation. Generally, radionuclide decay, γ-rays, high energy X-rays, and high energy particles (e.g., proton beams) must exhibit energies greater than 250 keV to result in Cerenkov radiation [56]. Early iterations of Cerenkov-radiation-induced PDT using radionuclides involved photoactivation of therapeutics [57] and activation of the intracellular PS protopophryrin IX [58]. Naturally, this process evolved into the use of Cerenkov-radiation-activable PNM systems that are activated by internal radionuclides or external high energy photons and particle beams that exceed the Cerenkov radiation threshold (Figure 8). Cerenkov radiation excites the PS which in turn results in the production of RMS. Apart from RMS, X-rays and γ-rays also lead to cell death as discussed above in the X-PDT section. Furthermore, radionuclides used for their production of Cerenkov radiation can kill the cell by directly damaging DNA in radionuclide therapy [59].

**Figure 9:**
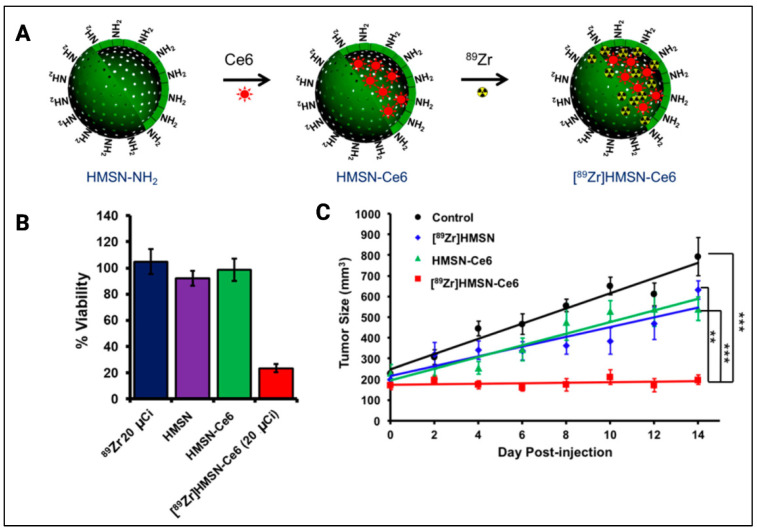
(**A**) A representation of the stepwise synthesis of the [^89^Zr]HMSN-Ce6 PNM complex for Cerenkov-radiation-induced PDT. (**B**) Cell viability of 4T1 cells when treated with ^89^Zr, HMSNs, HMSNs-Ce6, and [^89^Zr] HMSN-Ce6. (**C**) Tumor (mammary carcinoma) growth curves of different treatment groups: control group (black), [^89^Zr] HMSN (blue), HMSN-Ce6 (green), and [^89^Zr]HMSN-Ce6 (red), with *n* = 4. Error bars are SD. Statistical analysis was calculated by the student’s t test (***, *p* < 0.001; **, *p* < 0.01) [60]. Copyright (2016) American Chemical Society.

Kamkaew et al. used the PS chlorin e6 incorporated into hollow mesoporous silica nanoparticles (HMSN) labeled with the Cerenkov-radiation-emitting radionuclide ^89^Zr (Figure 9) [60]. This method achieved deep-tissue Cerenkov-radiation-induced PDT without the use of any external light sources. Breast cancer cells (4T1) were used to study the in vitro effects of the [^89^Zr] HMSN-Ce6 complex. [^89^Zr] HMSN-Ce6 caused more cell death than the control arms with a final cell viability of 20%. It was also shown that with increased concentrations of Ce6, more damage to the cells occurred. In vivo studies were performed on 4T1 tumor-bearing Balb/c mice with an average tumor size of 200mm^3^. Three different groups were included in the comparison: (1) [^89^Zr] HMSN-Ce6, (2) [^89^Zr] HMSN, and (3) HMSN-Ce6 (*n* = 4). The percentage reduction in tumor mass was calculated for each group. [^89^Zr] HMSN-Ce6 showed a 75% tumor reduction, [^89^Zr] HMSN showed a 20% tumor reduction, and HMSN-Ce6 showed a 32% tumor reduction when compared to the control group. In another study by Duan et al., dopamine-coated TiO_2_ nanoparticles were used as a photocatalytic PNM, and ^68^Ga-bovine serum albumin (BSA) served as the Cerenkov-radiation-emitting radionuclide [61]. The authors used them individually for inhibiting murine breast cancer models in vitro and in vivo and compared the Cerenkov effect of ^68^Ga-BSA and ^18^F-FDG (fluorine-fluorodeoxyglucose). It was subsequently found that TiO_2_-^68^Ga-BSA provided superior tumor suppression. In vitro studies on 4T1 cells showed four times more cell toxicity with a cell viability of less than 30% when treated with D-TiO2 (20 mg/kg, 50 μL) and ^68^Ga-BSA (30 MBq, 50 μL) than ^68^Ga-BSA alone. In vivo studies on 4T1 tumors in Balb/c mice showed that the group (*n* = 4) treated with ^68^Ga-BSA and D-TiO_2_ showed a significant reduction in tumor sizes of <100 mm^3^ compared to the control group treated with PBS alone (tumor volume >900 mm^3^) and increased the survival time when compared with control and other treated groups.

Wang et al. used ^131^I labeled zinc tetra(4-carboxyphenoyl) phthalocyaninate (ZnPcC4)-conjugated to a Cr^3+^-doped zinc gallate nanoplatform [62]. Through Cerenkov radiation, ^131^I can activate the PS ZnPcC4 and was found to be effective for deep-tissue PDT. In vitro studies were carried out using 4T1 cells, and MTT assays showed that the ^131^I-ZGCs-ZnPcC4 complex exhibited a lower cell viability of around 30% at a concentration of 40 μCi/mL I^131^ and 80 μg/mL of ZGCs-ZnPcC4 when compared to the control group. To quantify the production of ^1^O_2_, the authors used the probe 9,10-dimethylnathracene (DMA). The results showed a higher production of ^1^O_2_ with increase in the concentration of the radioactive Cerenkov-radiation-generating PNM ^131^I-ZGCs-ZnPcC4. In vivo experiments showed a reduction in tumor volume of around 200 mm^3^ after 14 d in groups treated with ^131^I-ZGCs-ZnPcC4 compared to the control which remained around 600–800 mm^3^.

Kotagiri et al. used TiO_2−_-based nano-PSs coated with polyethylene glycol for Cerenkov-radiation-induced PDT along with ^64^Cu (^64^Cu-TiO_2_-PEG NP) [63]. The authors used TiO_2_ coated with transferrin for intravenous administration and tumor tissue specificity. For the in vitro studies, human fibrosarcoma (HT1080) cell lines were used. Results showed that cells treated with transferrin-coated nanoparticles, along with the ^64^Cu radionuclide, exhibited the lowest cell viability of around 20% while other groups exhibited higher viability. To study the production of RMS through Cerenkov-radiation-induced PDT, the authors used hydroxyphenyl fluorescein (HPF) to quantify hydroxyl and peroxyl radicals and the Mitosox probe for superoxide anion radicals. Results revealed increases in fluorescence of both HPF and Mitosox probes when the cells were treated with ^64^Cu-TiO_2_-PEG NP, which confirmed the production of hydroxyl and superoxide anion radicals through Cerenkov-radiation-induced PDT. In vivo experiments demonstrated that the highest reduction in tumor volume was found when treated with TiO_2_-PEG NPS (2.5 μg/mL) and ^64^Cu (0.5 mCi/0.1 mL), which was <100 mm^3^ after 28 d, while tumors in other treatment groups were between 800 to 1000 mm^3^.

External beam radiation using energies above the Cerenkov radiation threshold have also demonstrated promise in activating PNMs. The study by Rossi et al. discussed in Section 2.3 demonstrated that porphyrin-conjugated nanowires can be activated with 6 MeV X-rays that are capable of generating Cerenkov radiation in tissue [38]. In this instance, it was difficult to delineate the contribution of Cerenkov-radiation-induced PDT from direct X-ray excitation of the PS and direct X-ray excitation of the nanowires that then activated the PS. Although no PNM was used, another important in vitro study leveraged γ-rays delivered by a Cs irradiator at 662 KeV (above the Cerenkov radiation threshold) to activate the PS pyropheophorbide, a methyl ester [64]. However, 10 µM of the PS and 58.5 Gy of γ-rays were needed to reduce A549 cell viability down to 20%, which was assessed by the CCK-8 assay. However, clonogenicity assays in the literature, which capture the radiobiological effects of γ-rays more accurately than the CCK-8 assay, reveal that A549 cell viability is less than 0.1% in response to only 10 Gy of γ-rays [65]. As such, the role of PS activation by Cerenkov radiation remains ambiguous at this time.

Cerenkov-radiation-induced PDT is one of the most significant and clinically relevant advances in deep-tissue PDT. However, Cerenkov-radiation-induced PDT using external beam radiation and radionuclides is not without its challenges. Cerenkov-radiation-induced PDT using external beam radiation suffers from the same limitations as radiotherapy, namely healthy tissue toxicity and fibrosis, as well as dosimetry challenges. With regards to Cerenkov-radiation-induced PDT using radionuclides, PNMs can provide a stable platform for targeted, co-delivery of both the PS and the radionuclide. However, the half-life of decay of radionuclides can often be significantly shorter than the time it takes for PNMs to accumulate in tumors. This will understandably compromise the efficiency of Cerenkov-radiation-induced PDT. Furthermore, PNMs co-delivering the PS and the radionuclide can be considered a constitutively active agent with a risk of disseminated and systemic phototoxicity wherever the PNMs accumulate. As such, unlike externally activated PDT, Cerenkov- radiation-induced PDT may not be safer than a more conventional “always on” chemotherapy regimen. A strategy to mitigate this risk is to separately administer the PS or radionuclide either in its free form or in PNM form. However, limited tumor tissue specificity of both agents can still be a limitation that might increase the risk of off-target phototoxicity.

### 2.5. Proton-Dynamic Therapy

Conventional external beam radiation therapy involves high energy photons while proton therapy uses accelerated proton beams. Protons lose energy upon electromagnetic interactions with electrons. When the protons reach lower energies, they rapidly deposit their remaining energy over a small focal region known as the Bragg peak. This Bragg peak is the therapeutic and cytotoxic window that needs to be spatially matched to the targeted cancerous lesion. The Bragg peak can be tuned to occur over a defined range within the tissue by selecting an appropriate span of initial proton energies. Thus, the depth of penetration of protons can be increased (or decreased) by amplifying (or reducing) the proton energy to include the target lesion in the Bragg peak [66]. For most clinical applications, combinations of the Bragg peak at different energies are used to provide a so-called spread-out Bragg peak (SOBP) to deliver a plateau-like dose distribution in order to treat the tumors. As such, proton therapy potentially lowers the radiation dose delivered to normal tissue, compared to external beam radiation therapy. Acknowledging the benefits of proton therapy, a role for using proton beams to activate PSs has emerged as proton-dynamic therapy, which was pioneered by the team of Theodossis Theodossiou.

A limited number of studies exist reporting proton-dynamic therapy. The first study by the team of Theodossiou reported the use of organic PSs in solutions or gels that were activated by accelerated protons as a proof of concept for proton-dynamic therapy [67]. Activation of the PSs by proton beams resulted in their fluorescence emission and generation of RMS that do not typically exist with proton therapy. The authors used three glioblastoma multiforme (GBM) cell lines (M059K, T98G, and U87) to demonstrate the in vitro efficacy of proton-dynamic therapy. The best overall effect was observed in the M059K cells with lower proton beam doses (2–10 Gy) while in T98G, the best effect was observed at higher proton beam doses (≥10 Gy). This is likely because the M059K cells are considerably more sensitive to proton therapy (even without PSs) than T98G. U87, on the other hand, did not produce any detectable proton dynamic cytotoxicity. Protons were accelerated by an MC-35 Scanditronix cyclotron (Scanditronix, Uppsala, Sweden) operating at an energy of 16 MeV. The relevance of this energy to the clinical cyclotrons will be discussed in Section 3. The mechanism of action of proton-dynamic therapy is speculated to be Coulombic excitation of the PSs as they are bombarded with protons. Considering the high energies of proton beams, it is also likely that Cerenkov radiation is involved in the PS activation process. The use of PNMs in proton-dynamic therapy is yet to be reported but holds considerable potential.

Recent studies have demonstrated the effectiveness of ultra-high dose rates of radiation, also known as FLASH radiation therapy (FLASH-RT). FLASH-RT delivers radiation doses at rates that are 10^4^-fold higher than conventional radiation therapy. Promising literature findings demonstrate that FLASH-RT reduces the toxic effects of conventional radiotherapy in healthy tissue, while maintaining tumor responses to external beam radiation [68]. The first evidence of FLASH-RT was demonstrated by Favaudon et al., where the authors used a linear electron accelerator for generating an ultra-high-dose rate beam (>40 Gy/s). They compared the efficiency of Flash dose rates (60 Gy/s) with the conventional dose rates (0.03 Gy/s) in the mice model of lung fibrinogenesis. Their results were that fibrosis was reduced when treating with FLASH-RT compared to conventional radiation therapy [69]. Though there is not much literature about proton-based FLASH therapy, it has been speculated that proton-based FLASH-RT can be a potential treatment for treating deep-seated tumors compared to conventional radiation therapy [70]. The significance of using FLASH-RT for deep-tissue PDT is that the Cerenkov radiation photon flux increases linearly with increasing dose rate of external beam radiation, without saturation [71]. While it has not yet been demonstrated for deep-tissue PDT, FLASH-RT using photon beams or proton beams has significant potential for activating PNMs in deeply seated tumors while synergizing tumor radiation damage and minimizing healthy tissue toxicity.

### 2.6. Chemiluminescence and Bioluminescence as FRET Donors for PDT

**Figure 10:**
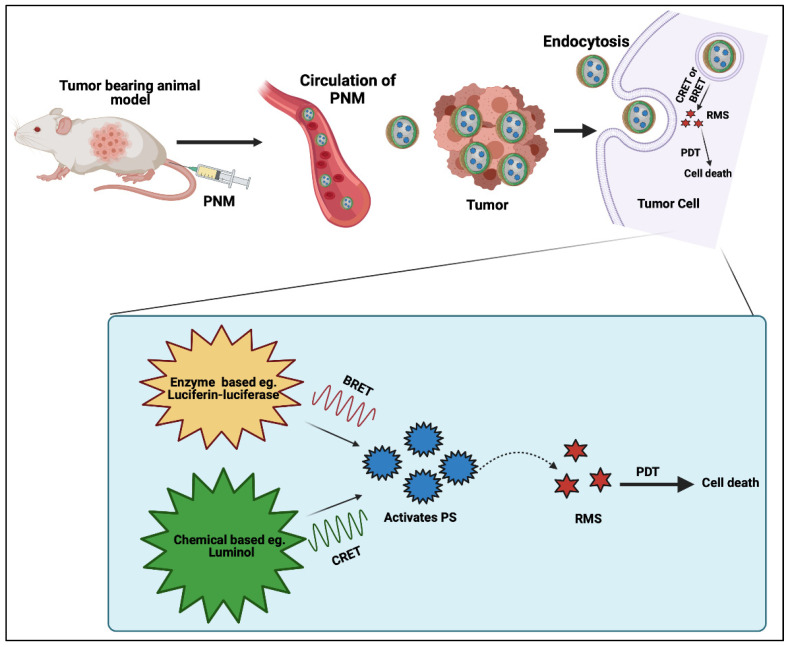
Schematic representation of chemiluminescence resonance energy transfer (CRET) and bioluminescence resonance energy transfer (BRET)-mediated PDT. (Figure is created from Biorender.com using an Academic License).

Self-illuminating systems, including chemiluminescent resonance energy transfer (CRET) and bioluminescent resonance energy transfer (BRET), have been used for various applications, including PDT. This self-illumination helps to activate the PS without the need for any external light source [72]. The distinction between CRET and BRET is in the origin of the substrates and analytes involved in the luminescence process. Bioluminescence occurs when an organism, such as certain insects and marine organisms, produces visible light through certain chemical reactions inside the body [73]. A common example of a bioluminescent producer is the luciferase enzyme. This process of endogenous luminescent activates PSs and helps to produce reactive molecular species to inhibit tumor cells [74]. Another self-illuminating system is chemiluminescence, which generally includes the production of light through a chemical reaction. This chemical reaction takes place between hydrogen peroxide found in cancer cells and higher energy compounds which can activate PSs [75]. An example of chemiluminescence is the reaction between nanoparticles containing luminol and horseradish peroxide with hydrogen peroxide. Figure 10 shows a schematic representation of CRET- and BRET-mediated PDT.

**Figure 11:**
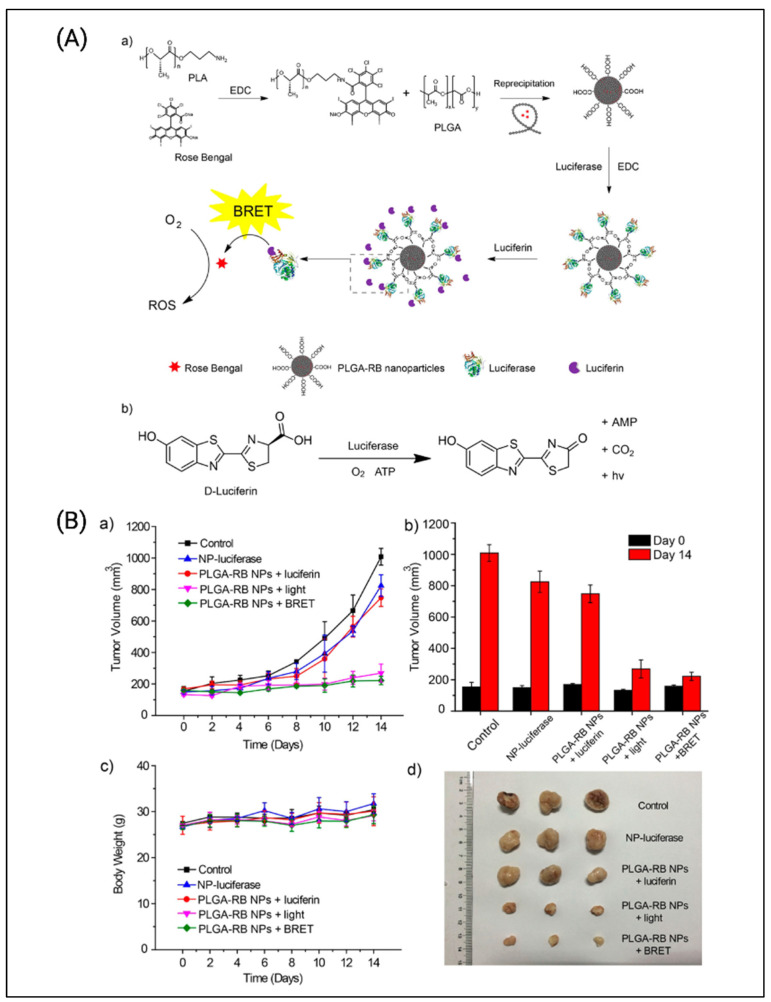
(**A**) A schematic representing the synthesis of PLGA-RB nanoparticles and mechanism of ROS generation following BRET. (**B**) Data from in vivo animal studies of the PLGA-RB nanoparticles by BRET-PDT. (**B**(**a**)) Tumor (mouse hepatocellular carcinoma) volume of 5 groups. Data are shown as the mean ± SD. (**B**(**b**)) Tumor volumes from 0 and 14 d. (**c**) Body weight of animals in all 5 groups. (**d**) Images of tumors excised on 24 d after treatment. (PLGA RB nanoparticles (PLGA-RB NPs): 40 μg/mL, PLGA-RB NPs conjugated with 20 μg/mL luciferase (NP-luciferase), luciferin: 60 μmol/L.) Reprinted and adapted with permission from [74]. Copyright (2018) American Chemical Society.

As shown in Figure 11, Yang et al. used PLGA (poly(lactic-co-glycolic acid)) nanoparticles, along with the PS rose bengal, with a firefly luciferase-based (BL-PLGA RB) bioluminescent system for BRET-PDT [74]. Luciferases generate bioluminescent signals in the presence of their substrate, luciferin, which was used to activate the PS rose bengal. The tumor volume of the group treated with BL-PLGA RB exhibited the lowest tumor volume of around 200 mm^3^ compared with the control which exhibited a higher tumor volume between 800 to 1000 mm^3^. Another study by Hsu et al. used Renilla luciferase along with quantum dots for BRET-PDT [73]. This conjugate of QD-RLuc8 exhibits bioluminescent emission at 655 nm once coelenterazine is added, which activates Foscan-loaded micelles for PDT. A549 cells were used for in vitro studies. The QD-RLuc8 BRET-PDT group exhibited the highest cytotoxicity with cell viability of around 50%. In vivo analysis revealed a lower tumor volume of around 200 mm^3^ with the BRET-PDT when compared with control arms, which demonstrated tumor volumes of around 500 mm^3^.

Wu et al. designed a self-luminescing CRET-PDT nanosystem denoting it as POCL [72]. The authors used bis[3,4,6-trichloro-2-9pentyloxycarbonyl) phenyl] oxalate (CPPO) which has high reactivity with H_2_O_2_ and acts as a power source with poly[(9,9’-dioctyl-2,7-divinylene-fluorenylene)-alt-2-methoxy-5-(2-ethyl-hexyloxy)-1,4-phenylene] (PFPV) as a converter of chemiluminescence and tetraphenyl porphyrin (TPP) as a PS. The authors encapsulated all three compounds in PEG-PCL/folate-PEG-cholesterol micelles. This system depends on the internal transfer of energy between the particle system from CPPO, which is relayed by PFPV and received by TPP to generate reactive molecular species. The greatest cytotoxicity of HeLa cells had a cell viability of around 38% following CRET-PDT using POCL compared to the control group. Mice with HeLa tumors were injected every 3 d for a total of 9 d with 100 μL of POCL/FA at a 0.2 mg/mL concentration. Results showed a reduction in tumor weight in CRET-PDT treated mice to less than 0.2 g compared to the control group which had a tumor weight of 0.8 g after 21 d. Chen et al. used 4, 4′-9-dibenzo [a, c] phenazine-9,14-diyl0 pyridin-1-iumbromide (DPAC-S) and cucurbit [7] uril (CB [7]) together as a supramolecular assembly for CRET-PDT [76]. The authors then co-assembled CPPO into the complex, which was endocytosed within cancer cells, to specifically target the mitochondria. The interaction between H_2_O_2_ and the complex resulted in chemiluminescence. In vitro comparisons between normal and cancerous cell lines (293T and KYSE-150, respectively) when treated with DPAC-S@CB [7] @CPPO showed that the complex showed no cell toxicity with 100% cell viability in the normal cell line while the cancerous cell line exhibited 80% cytotoxicity when treated with the complex DPAC-S@CB [7] @CPPO (2 × 10^−5^ M) and H_2_O_2_ (1 mM).

The lack of a need for an external activation step for PNMs is an attractive facet of CRET- and BRET-PDT. However, such systems are complex and often require separate administration of substrates and analytes. This complexity is likely to hamper clinical translation; however, CRET- and BRET-PDT have significant potential for eradicating deeply seated tumors, especially disseminated micrometastases, and warrant further investigation. BRET-PDT especially will require the careful engineering of recombinant bioluminescence enzymes with the lowest degree of immunogenicity.

### 2.7. Sonodynamic Therapy

**Figure 12:**
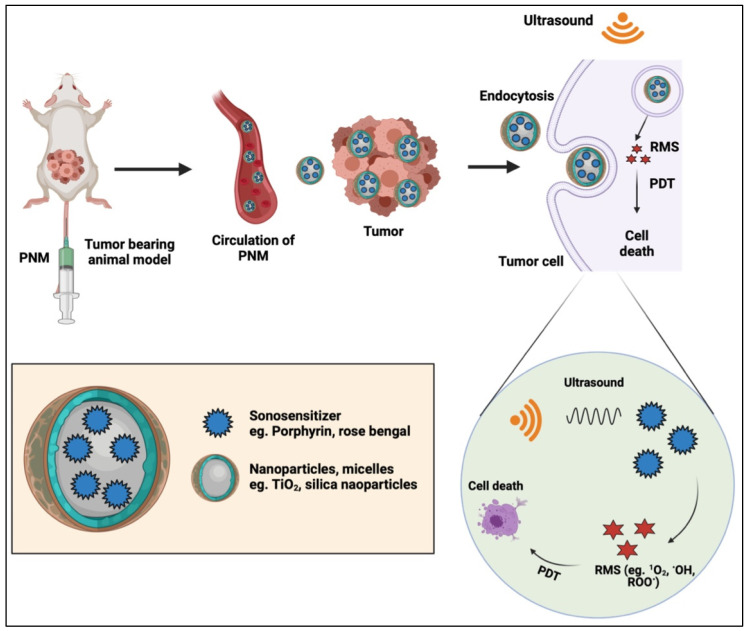
Schematic representation demonstrating the processes behind sonodynamic therapy (SDT) using PNMs. (Figure is created from Biorender.com using an Academic License).

Sonodynamic therapy (SDT) is a highly promising modality that has recently entered clinical trials for gliomas (NCT04845919, NCT05123534, NCT04559685). SDT was developed from the early discovery that ultrasound fields can activate PSs, which are termed sonosensitizers in the context of SDT [77,78,79]. This approach was first explored in 1989 by the team of Yumita, who showed that the PS hematoporphyrin can be used to generate cytotoxic effects in acoustic fields [77]. SDT can be triggered using low-intensity focused ultrasound without or with microbubble cavitation and other acoustic wave-based therapies. Different sonosensitizers can be formulated into PNMs, and most commonly include 5-aminolevulinic acid-induced protoporphyrin IX, rose bengal, and chlorin e6 [72,73] The mechanisms underlying SDT using PNMs specifically are a combination of cavitation-induced activation of formulated sonosensitizers, nanoparticle-enhanced cavitation, enhancement of mechanical membrane damage, microstreaming, and potentiation of cavitation-induced pyrolysis of water [80]. Figure 12 shows a schematic representation of the process behind SDT while Table 3 shows a summary of various types of sensitizers used to generate reactive molecular species with different ultrasound parameters [81].

McEwan et al. developed a strategy to treat hypoxic tumors using oxygen-carrying microbubbles using SDT [82]. The authors conjugated microbubbles containing oxygen with rose bengal for SDT in a pancreatic cancer model. McEwan et al. also compared these models with sulfur hexafluoride microbubbles and assessed their effectiveness using in vitro and in vivo models. In vitro analysis was performed using the BxPC3 pancreatic adenocarcinoma cell line. The cells were treated with microbubbles and rose bengal along with ultrasound. The results indicated that cells treated with microbubbles, rose bengal and ultrasound exhibited cell viabilities <60% when compared to control cells. For in vivo analyses, mice (*n* = 3) with BxPc-3 tumors were administered with 60 μL containing 1.5 × 10^7^ microbubbles and 91 μM of RB through injection. Following microbubble administration, ultrasound irradiation was performed at a frequency of 1 MHz and a power of 3.5 W/cm^2^ for a total of 3.5 min. The authors demonstrated that mice treated with oxygen-microbubble-rose bengal conjugates, along with ultrasound exposure, showed a 45% reduction in tumor volume, compared to mice treated with only conjugates after 5 d of the treatment. Borah et al. used HPPH ([3-(1-hexyloxy) ethyl-3-devinyl-pyropheophorbide-a)) as both a PS and sonosensitizer [83]. To facilitate solubility and tumor delivery, the authors formulated HPPH in cationic polyacrylamide nanoparticles. In vitro cells incubated with the nanoparticles were exposed to ultrasound for 60 min at 0.5 W/cm^2^. Results demonstrated higher cytotoxicity in the group treated with SDT for 60 min compared to the PDT only group and the control. Cell viability in the SDT+PDT group was 40%, while the PDT only showed more than 80% viability. For in vivo studies the authors used a mice model of malignant glioma and administered it with HPPH after loading it into the cationic nanoparticles at a therapeutic dose of 0.47 μmol/kg. For PDT, mice were treated with a light dose at 655 nm after 24 h of injection at an irradiance of 75 mW/cm^2^. For SDT, the authors used ultrasound at 0.5 W/cm^2^ with 3 MHz for 30 min. Initial tumor reduction was greater than 50% in the treated group. Using this method, the authors demonstrated the synergistic effect of using both light-activated PDT and ultrasound-activated SDT.

Acoustic waves are advantageous in that their penetration depths exceed visible and NIR light, and, unlike external beam radiation and radionuclides, acoustic waves are nonionizing. Of all deep-tissue activation mechanisms to be used in conjunction with PNMs, SDT is the closest to clinical adoption considering that patients are already in trials for SDT. The limitation of PNMs for SDT, however, are largely centered on the complex and poorly defined mechanisms of action. Further in-depth studies into the mechanisms of action and fate of PNMs after ultrasound activation are likely to increase confidence and expedite translation.

**Table 3:** Summary of different types of sensitizers used to generate reactive molecular species following activation with ultrasound at various frequencies and intensities.

Sensitizer	Reactive Molecular Species Detected	Ultrasound Frequency (MHz)	Ultrasound Intensity (W/cm^2^)	Exposure Time	Reference
Protoporphyrin IX (using the precursor 5-Aminolevulinic acid)	n.a.	1.04	10	5 min	[84]
Acridine Orange	^1^O_2_ + ^•^OH	2	2.0	60 s	[85]
Chlorin e6	^1^O_2_ + ROO^•^	1.56	6	3 min	[86]
C1A1-phthalocyanine	n.a.	3	6.0	60 s	[87]
DCPH-P-Na(I)	^1^O_2_	1	0.5 to 2.0	10 min	[88]
Hematoporphyrin	^1^O_2_	1	1	120 s	[89]
Hypocrellin SL052	n.a.	1	0.4 to 0.8	3 min	[90]
Indocyanine green	n.a.	1	3.5	3 min	[91]
Methylene blue	^•^OH	2	0.24	30 s	[92]
Photofrin	^1^O_2_	1	0.5	2 min	[93]
Phthalocyanine	n.a.	1	2	10 min	[94]
Protoporphyrin IX (using the precursor 5-Aminolevulinic acid)	^1^O_2_	1	0.5	15 min	[95]
Rose bengal	^1^O_2_	1	1	5 min	[96]
Rose bengal derivative	^1^O_2_	1.92	8.3	60 s	[97]

## 3. Capitalizing on Clinically Available Technologies for Deep-Tissue Activation

Currently, there are a variety of direct PDT photoactivation systems available. For example, the Modulight ML7710-PDT Laser System has been used for abscess cavities located deep in human tissue (NCT02240498). The system consists of a control module used to apply the desired laser settings connected to an optical fiber which is inserted into the body. The system supports laser wavelengths of 400 to 2000 nm, and the optical output power typically used with the device in PDT ranges from 1 to 15 W. While direct PDT can be somewhat limited by the penetrative ability of visible light for direct excitation, as discussed in Section 1 above, it is possible to bypass this drawback. The general preclinical range for direct PDT is between 100 to 400 mW/cm^2^. For cases when direct PDT is feasible, the Modulight system provides the technology to do so. A list of examples of other clinical systems for direct PDT is shown in Table 4.

Although not used in the clinic to date, two-photon PDT has clinical potential in treating cancer. The MPTflex and MPT DermaInspect are two multiphoton tomographs currently used for two-photon diagnosis of skin diseases. These devices both have a range of 720 to 920 nm and repetition frequency around 80 MHz but differ in their power output; the MPTflex cannot exceed 50 mW while the DermaInspect can reach 1.5 W [97]. Current clinical applications of two-photon excitation have primarily used the lower energy output of the MPTflex for imaging of the skin. However, to excite PNMs for therapy within deeper tissues, a higher energy will be needed, such as that of the DermaInspect. More information on examples of two-photon PDT energies and doses used clinically can be found in Table 5.

Upconversion PDT utilizes the previously discussed medical research technologies used in traditional PDT and two-photon PDT but focuses on the optimal optical transparency of tissue of 700 to 1100 nm in the near-infrared range of light, which allows for deeper penetration of tissue compared to visible light. The previously discussed systems have operating ranges within this optimal optical transparency range, allowing for these traditional and two-photon PDT methods to serve a dual purpose of utilizing upconversion PDT as well. Many devices containing 980 nm lasers that are used in the clinic can also be used for upconversion PDT, such as the Velure S9, which is used commonly in dental procedures. This device is used at power up to 2.5 W/cm^2^ and UCNPS in the preclinical setting are typically used at powers up to 2 W/cm^2^, making clinical translation of upconversion PDT feasible [103,104]. The general preclinical range for upconversion PDT is 0.5 to 2.5 W/cm^2^. A list of technologies used in the clinical settings that can be used for upconversion PDT are summarized in Table 6.

X-ray instrumentation is among the most widely clinically available medical technologies. One example of a common X-ray instrument is the Siemens YSIO X.pree model imaging system. It operates up to 150 keV with the X-ray generator operating at 65 and 80 kW. According to the manufacturer, the testing X-ray dosage was 2 µGy, but dosages may be increased or decreased depending on the personnel operating the device. With the potential of operating up to 150 keV, this already clinically available X-ray irradiation device can be tuned to directly excite various PSs and allow for X-PDT [107] The safety of such an approach would have to be assessed carefully.

Another example of a clinically available X-ray irradiation device is the Varian TrueBeam LINAC, which has been used pre-clinically for X-PDT. In contrast to the Siemens YSIO X.pree model, the TrueBeam has the potential to operate in the MeV range and has already been used in radiotherapy studies at 6 and 10 MeV energies. As shown in Table 1, the PSs induced by X-ray irradiation in preclinical research require varying energies and doses, but the energies generally range from 75 to 100 keV and an operation dose of approximately 5 Gy. Thus, the Siemens YSIO X.pree model, while it can operate at an appropriate energy, will require a significantly higher dose than is traditionally administered and will therefore require prolonged irradiation times. Meanwhile, the Varian TrueBeam operates at a much higher energy than is usually associated with X-PDT and thus, will require further testing for PNM excitation [107,108]. The general preclinical range for X-PDT is between 75 to 220 keV (Table 1). A summary of clinical devices that are currently used in X-ray irradiation is provided in Table 7.

In terms of penetration potential, γ-ray devices used in radiotherapy can also potentially be used to apply deep-tissue Cerenkov-radiation-induced PDT. The Lars Leksell Center for Gamma Surgery at the University of Virginia includes an Elekta Leksell Gamma Knife Perfexion, a medical device that utilizes a cobalt-60 source for a focused beam of γ-irradiation. The decay of cobalt-60 produces an electron with an energy up to 315 keV and two 1.17 and 1.33 MeV γ-rays [112,113]. Since the energy exceeds the Cerenkov radiation threshold, it is conceivable that γ-ray radiotherapy can be used in the clinic to enable deep-tissue PDT. Further information on the γ-ray instrumentation available in the clinic is summarized in Table 8.

Proton therapy is another method used to treat cancer that has been shown in vitro to have potential to activate PNMs for proton-dynamic therapy. This approach can synergize proton therapy with proton-dynamic therapy, thereby potentiating the effect of the applied dose. The IBA cyclotron is a widely used proton beam irradiator for cancer treatment, which can generate up to 231 MeV proton beams. One study utilized the IBA cyclotron to produce proton beams ranging from 100 to 150 MeV energies with a dosage of 30 to 55 Gy [114,115]. Another example of a clinically available cyclotron is the Mevion S250 Proton Therapy System, a device capable of producing proton beams ranging from 1 to 250 MeV. With the ability to excite different PNMs over a broad energy range, the Bragg peak could be tuned with this device to achieve successful proton-dynamic therapy. Preclinical evidence of proton-dynamic therapy typically uses energies in the 10 to 20 MeV range with a dose between 2 and 20 Gy. As such, the Mevion S250 Proton Therapy System could be readily adopted to bring proton-dynamic therapy to the clinic without difficulty [116]. More information on two examples of clinical devices that could be used for proton-dynamic therapy can be found in Table 9.

Sonodynamic therapy using PNMs is another application of PDT that could be achieved through ultrasound devices already used clinically. Emerging PNM activation strategies could capitalize on currently available high-intensity focused ultrasound systems that are already used for SDT in the clinic. The Holologic Viera Portable Breast Ultrasound device operates at frequencies between 4 to 14 MHz with 1 to 20 MHz transmission. This manufacturer also fabricates multiple other ultrasound devices, many of which include 1 MHz in their frequency ranges, such as the Clarius HD PA system [116]. Another ultrasound system used for non-cancer SDT of atherosclerotic lesions in the First Affiliated Hospital of Harbin Medical University, Harbin, China uses an intensity of 1.6 W/cm^2^ with a resonance frequency of 1 MHz (NCT03871725). Given the availability of ultrasound systems in the clinic that operate within the frequencies and intensities that can activate a broad range of PS molecules (0.5 to 10 W/cm^2^ intensities and ca. 1 MHz resonance frequencies; Table 3), it is conceivable that current clinical ultrasound systems can be extended to PNM activation. High intensity focused ultrasound (HIFU) devices have been explored for tumors as a nonionizing form of cancer treatment with frequencies ranging from 0.5 to 8 MHz. The Model-JC HIFU system developed by the Chongqing HAIFU Company in Chongqing, China, is one such example used in HIFU research. It was found that frequencies around 1 MHz have been most useful for heat deposition, which aligns relatively well with the activation frequencies of PSs potentially useful in SDT [119]. As such, a combination HIFU-SDT treatment is also worth exploring in future studies using promising, emerging PNM platforms. One limitation is the focused area of treatment of existing FDA approved HIFU systems, precluding diffuse treatment of invasive disease. Further information on a number of clinical ultrasound devices that can be used for PNM activation can be found in Table 10.

Although direct photoexcitation devices specifically used for PDT already exist, a plethora of other clinically used medical devices are well established in clinical practice and can be repurposed as alternative energy sources for deep-tissue excitation of emerging PNMs. A critical aspect of using alternative energy sources for deep-tissue activation of PNMs is the potential for therapeutic synergy between the alternative energy source (e.g., X-rays, proton beams, HIFU), and deep-tissue PDT. Furthermore, relevant alternative energy sources could also be used as simultaneous imaging modalities for image-guided deep-tissue PDT.

## 4. Perspectives

The vast majority of deep-tissue-activated PNM treatments remain in the preclinical setting. Although the growth of emergent nanotechnology approaches for deep-tissue-activable PNMs is still ongoing, no clear path to clinical translation has emerged. The reason for this delay is two-fold: (1) the complexity of the material constituents of PNMs, and (2) the lack of clinical instrumentation available in the preclinical setting to demonstrate efficacy and optimize PNM design.

While the complexity of material constituents is a limitation for all nanomedicines in development for human use, the most direct path to clinical translation of deep-tissue-activable PNMs involves the use of approved nanoformulations (e.g., micelles, liposomes, polymer carriers) as templates. Although this precludes a significant proportion of deep-tissue-activable PNMs that comprise complex (in)organic nanomaterials, these materials are being gradually introduced into the clinic as simplified precursors. As their safety is better understood, and innovations in physiological clearance (e.g., renal clearance) become more controlled, such complex (in)organic deep-tissue-activable PNMs may eventually find a niche in the clinic.

The focus here, however, has been on how some deep-tissue PNM activation protocols may be achieved using pre-existing clinical instruments. Considering that PDT (as well as deep-tissue PDT) is primarily considered as a drug-device combination by regulatory agencies throughout the world, translation can be drastically expedited by repurposing instrumentation that is already in clinical use for other indications and purposes to activate PNMs for deep-tissue PDT. A number of these, including NIR and IR light, ultrasound and ionizing radiation are already well established in the clinic, and hold the greatest potential. However, there may be added layers of complexity when adopting these activation strategies for PNM activation and deep-tissue PDT. For example, radiation dosimetry has been well-established by several years of clinic experience with positive and consistent outcomes. The optimal combinations of radiation dosimetry and radiation-induced PDT dosimetry can be particularly challenging to determine due to the added complexity of PS and PNM dosimetry. It is conceivable that PDT will play the most important role in areas where organs are at risk due to their close proximity to tumors, and typical radiation doses would inevitably introduce normal tissue toxicity. An example of such a scenario would be the risk of radiation damage to the duodenum when irradiating pancreatic tumors, or the risk of radiation damage to the optic chiasm when irradiating brain tumors. If radiation-activated PDT, such as X-PDT, can facilitate tumor control while lowering the radiation dose needed, and while reducing toxicity at a particular site, this approach may have the greatest impact.

By focusing the development of deep-tissue-activable PNMs on simpler, more clinically translatable materials, in addition to identifying the best suited pre-existing clinical instrumentation that can be leveraged for their activation in patients, a clearer path to translation can be paved. In doing so, the remarkable efficacy demonstrated in preclinical models for PNMs activated without an external light source can be delivered to patients at a faster rate to provide the greatest impact on cancer patient treatment outcomes, well-being, and survival.

## 5. Conclusions

It is evident that transformative technologies, such as PDT, are becoming increasingly valuable enabling technologies for frontline therapies, such as chemotherapy, radiotherapy and immunotherapy. By inducing tumor tissue photodamage and priming the tumor tissue for improved drug delivery, enhanced immune responses and synergistic combination regimens, PDT using nanotechnology PNMs is playing a central role in treating solid tumors. The use of a PNM platform has paved the way for integrating nanomaterials capable of being excited by alternative energy sources that penetrate deeper into tissue than visible-NIR light to actualize deep-tissue PDT. These alternative energy sources can in and of themselves be therapeutic, such as X-rays, thereby facilitating a rational synergy between a standard of care modality and deep-tissue PDT. Imaging modalities, such as ultrasound, can also be leveraged as an alternative energy source, thereby facilitating image guidance for treatment planning and response monitoring. As safer and more predictable deep-tissue-activable nanomaterials are developed, and a clearer vision of clinical instrumentation that can be adopted, deep-tissue activation of photonanomedicines will undoubtedly draw nearer to clinical transition. Thus, deep-tissue PDT using translatable PNMs may then address a critical unmet clinical need to provide rational, synergistic treatment of deeply seated, irregular, invasive and metastatic lesions and provide innovative options for patients who would not otherwise respond to frontline standard of care therapies.

## Figures and Tables

**Table 1:** Clinical examples of inorganic and organic precursors of optically active photonanomedicines (PNMs) explored preclinically for deep-tissue PDT.

PNM Precursor	Respective Class of PNM Used Preclinically	Example of Clinical Trial Using PNM Precursor	Clinical Utility	Clinical Format
Gadolinium nanoparticles	X-ray scintillators	NCT04899908	Treating patients with brain metastases	Gadolinium-based nanoparticles
Zirconium complexes	Cerenkov-activable PNMs	NCT04758780	PET/CT imaging in metastatic triple negative breast cancer	^89^Zr-labeled girentuximab
Titanium dioxide nanoparticles	Cerenkov-activable photocatalytic PNMs	NCT03250520	Delivery vehicle for platinum acetylacetonate chemotherapy	Platinum acetylacetonate (1%wt) supported by sol-gel technology functionalized with titania platinum
Gallium complexes	Radionuclide labeled, Cerenkov-activable PNMs	NCT04023240	PET/CT imaging of cancer-associated fibroblasts	^68^Ga-FAPI (68-Ga-Fibroblast activation protein inhibitor)
Ruthenium complexes	Two-photon excited PNMs	NCT03945162	PDT of non-muscle invasive bladder cancer	TLD-1433 Ruthenium complex
Gold nanoparticles	X-ray radiosensitizers	NCT04240639	Photothermal therapy of prostate neoplasms	Gold-coated silica nanoshells
Renilla luciferase	Bioluminescence resonance energy transfer-activable PNMs	NCT00794131	Safety evaluation of vaccina virus in patients with solid tumors	GL-ONC1 a genetically engineered vaccina virus encoding Renilla luciferase

**Table 2:** A summary of the different energies and respective PSs and PNMs used for deep-tissue PDT with X-ray activation, along with the specific RMS detected.

Energy of Applied X-rays (keV)	Dose of Applied X-rays (Gy, If Mentioned)	Reactive Molecular Species Detected	PS (Scintillating/Vehicle Nanoformulation)	Reference
75	1	^1^O_2_	rose bengal (glutathione-protected gold nanoclusters)	[40]
15	n.a.	^1^O_2_	hypericin (GdEuC1_2_ micellar particles)	[41]
75	n.a.	^1^O_2_	rose bengal (mesoporous LaF_3_:Tb nanoscintillators)	[42]
160	5	^1^O_2_	rose bengal (mesoporous silica nanoparticles with NaLuF_4_: Gd^3+^,Eu^3+^,Tb^3+^)	[43]
50	5	^1^O_2_	2,3-naphthalocyanine (mesoporous silica nanoparticles with LiGa_5_O_8_: Cr)	[44]
220	8	^•^OH	zinc oxide nanoparticles (Ce(III)-doped LiYF_4_ core-shell nanoscintillator)	[45]
225	2	^1^O_2_	iridium and ruthenium complexes (Hf_6_O_4_ -based metal-organic layers)	[46]
50	1 to 10	^1^O_2_	merocyanine 540 (mesoporous silica coated SrAL_2_O_4_: Eu^2+^ nanoscintillators)	[36]
120	2	^1^O_2_	tetrabromorhodamine-123 (copper and cobalt co-doped ZnS)	[47]
90	3	^1^O_2_	protoporphyrin IX (PLGA microspheres with Cerium (III)-doped lanthanum (III) fluoride (LaF_3_: Ce^3+^)	[48]
44	11	^1^O_2_, ^•^OH	porphyrin (polysiloxane layered Tb_2_O_3_ nanoscintillators)	[49]
6000	0.4 to 2	^1^O_2_	porphyrin (SiC/SiOx nanowires)	[38]

**Table 4:** Examples of currently used instrumentation in clinical use for direct PDT along with their typical dose parameters.

Manufacturer	Model	Clinical Operating Powers	Clinical Utility	Typical Wavelengths and Dose Parameters	Reference
Modulight	ML7710-PDT laser system	1 to 15 W	For sterilization of deep tissues	668 nm, 20 mW/cm^2^	NCT02240498 [98]
MMOptics	Laser Duo^®^ red laser diode	100 mW	Treatment of Herpes labialis	660 nm, 300 J/cm^2^, 3 J at center of lesion	NCT04037475
BIOSPEC	UFPh-675-01-BIOSPEC	1000 mW top optical power	Anti-viral treatment for COVID-19	650 nm, 36 J/cm^2^	NCT04933864 [99]
Diomed Inc, An-dover, MA	InGaAIP laser diode	2000 mW maximum power	Treatment for advanced rectal cancer	633 ± 3 nm, 200 J/cm^2^	NCT01872104
PDT^®^ HGesmbH, Vienna, Austria	30 or 70 mm radial light applicator	n.a.	Treatment for malignant biliary obstruction	650 nm with 400 mW/cm^2^ and 250 J/cm^2^ during 650 s radiation time	NCT02504957 [100]

**Table 5:** Examples of two-photon imaging instruments in clinical use along with typical parameters and operation ranges. These systems can be readily adapted to achieve two-photon PDT in patients.

Manufacturer	Model	Clinical Operating Powers	Clinical Utility	Typical Wavelengths	Reference
MPT	DermaInspect	0 to 1.5 W	Diagnosis of dermatological disorder	720 to 920 nm	[101]
MPT	MPTflex	2 to 50 mW	Diagnosis of dermatological disorder	710 to 920 nm (45 mW:5 mW pump beam:Stokes beam)	[102]

**Table 6:** Examples of clinically used lasers and details of the typical dose and operation ranges, which can be adapted for upconversion PDT.

Manufacturer	Model	Clinical Operating Powers	Clinical Utility	Typical Operation Ranges	Reference
Lasering Medical Laser	Velure S9/1064	0.5 to 50 W	Treatment of chronic periodontitis	980 nm, n.a.	[105,106]
A.R.C. Laser	Chirolas A.R.C. Laser	Up to 20 kW	Laser assisted frecnectomy	980 nm, 8 kW/cm^2^	[103,104]

**Table 7:** Examples of clinically used X-ray instruments with their operation ranges and typical dose for tissue imaging and radiation therapy.

Manufacturer	Model	Clinical Operation Ranges	Clinical Utility	Reference
Siemens	YSIO X.pree	Up to 150 KeV, 65 kW, 85 kW^2^	Diagnosis and therapeutics	[107,108,109]
Varian	TrueBeam	6 and 10 MeV	Radiation therapy for body tumors	[110,111]

**Table 8:** Example of currently used instrument for γ-ray radiotherapy along with its operation energies in the clinic.

Manufacturer	Model	Clinical Operating Energies	Clinical Utility	Reference
Elekta	Leksell Gamma Knife Perfexion	Co^60^ decay simultaneously produces combination of: one 315 keV photon, one 1.17 MeV γ-ray and and 1.33 MeV γ-rays	Treating brain tumors	[112,113]

**Table 9:** Examples of cyclotrons used for proton therapy along with their typical doses, operation ranges and clinical utilities.

Manufacturer	Model	Operation Ranges	Clinical Utility	Typical Dose Parameters	Reference
IBA (Belgium)	n.a.	100 to 231 MeV	Treatment of central nervous system germ cell tumors	30 to 55 Gy	NCT01049230 [114,115]
Mevion	S250 Proton Therapy System	1 to 250 MeV	Treatment of intracranial tumors	2 Gy fractions up to 400 Gy	[117,118]

**Table 10:** Examples of clinical instruments for ultrasound imaging and therapy along with their typical operation ranges and further details of parameters when used in the clinic.

Manufacturer	Model	Operation Ranges	Clinical Utility	Parameter Details	Reference
Holologic	Viera Portable Breast Ultrasound	4 to 14 MHz	Ultrasound sonography of breast	1 to 20 MHz waveform transmission	[116]
Chongqing HAIFU™ Company, Chongqing, China	Model-JC High-Intensity Focused Ultrasound (HIFU) system	650 and 800 W	HIFU treatment of various solid tumors	No dose limit	[119]
Insightec, Haifa, Israel	ExAblate 4000 Type II Neurosystem	620 to 720 kHz	Magnetic resonance (MR)-guided focused ultrasound System sonodynamic therapy of gliomas	5 to 60 s pulse duration	NCT04845919
